# ‘A tool we need’: Midwives’ descriptions and recommendations of an ideal obstetric triage tool

**DOI:** 10.4102/hsag.v27i0.2029

**Published:** 2022-10-26

**Authors:** Kagiso P. Tukisi, Annie Temane, Anna Nolte

**Affiliations:** 1 Department of Nursing Science, School of Health Care Sciences, Sefako Makgatho Health Sciences University, Pretoria, South Africa; 2 Department of Nursing, Faculty of Health Sciences, University of Johannesburg, Johannesburg, South Africa

**Keywords:** obstetric triage tool, ideal obstetric triage tool, description, recommendations, midwives

## Abstract

**Background:**

The obstetric triage tool (OBTT) is used to record the clinical findings following obstetric triage (OBT). The recorded OBTT provides midwives with clinical information leading to diagnosis of existing and potential maternal and foetal problems that may lead to intrapartum complications, planning of specific midwifery care and communication among the midwifery team about the woman in labour.

**Aim:**

This study aimed to explore and describe midwives’ experiences of the OBTT used during admission of women in labour in the Bojanala district.

**Setting:**

This study was conducted in the two selected facilities in Bojanala district in North West province.

**Methods:**

This study is a derivative of a major study, entitled ‘Midwives’ experiences of OBT by midwives in the Bojanala district’. A qualitative, explorative and descriptive research design was followed. Nine purposefully sampled midwives with over 5 years of clinical midwifery experience, employed in the Bojanala district, attended a semistructured interview. Data obtained were analysed using Colaizzi’s descriptive method of data analysis according to the themes and categories which emerged.

**Results:**

One central theme with 10 subthemes emerged. Midwives verbalised their dissatisfaction with the current OBTT and made recommendations for the revision of the tool.

**Conclusion:**

The study highlighted midwives’ experiences of the OBTT and recommendations for an ideal tool based on their knowledge of admission of a woman in labour.

**Contribution:**

This study provides a new OBTT from midwives’ perspectives that could be useful in improving pregnancy and labour outcomes in clinical midwifery practice.

## Introduction

Obstetric triage (OBT) is a systematic process to comprehensively gather clinical data about the woman in labour as well as the foetus upon admission, with the aim of prioritising the woman based on her health needs (McCarthy, Pollock & McDonald 2021). The findings of OBT are recorded on the obstetric triage tool (OBTT), which refers to a clinical tool designed for standardising the OBT care rendered by midwives and obstetricians to pregnant women admitted to the labour ward (Rashidi Fakari et al. 2019). The South African Nursing Council (SANC 2014) provides a clear prescription that recording is an integral part of midwives’ responsibilities. This renders an OBTT a legal document in the midwifery settings. Despite the fact that recording is an integral part of midwifery practice, clinical records audits revealed that cases where notes are missing or insufficient are still reported (Vallely et al. 2020).

The contents of the OBTT aid midwives in drawing clinical judgements and justify their clinical decisions and actions during OBT. An OBTT should provide the following spaces for clinical information. This information includes physical examination and vital signs such as blood pressure, pulse, temperature, respiration and urinalysis (Kodama et al. 2021). These details are diagnostic of ongoing pregnancy and labour-related complications and are an important part of the OBTT (Kodama et al. 2021). Findings of history-taking are to be recorded as they aid in establishing the present history related to labour. The maternal history guides the attending midwives to probe further to reveal the pre-existing medical conditions such as pre-eclampsia, which will warrant a far more specific and vigilant intrapartum care because of the complications exacerbated by the process of labour (Department of Health [DOH] 2016). Abdominal examination findings guide the midwives on foetal size in relation to estimated gestational age, which would help guide the management for low- and high-risk labour (DOH 2016). Admission cardiotocograph (CTG) findings serve as a baseline for foetal well-being, and the possibility of foetal distress can be anticipated early in labour (Devane et al. 2017).

An OBTT as a clinical record serves as a means of communication among the midwifery team during handover of patients (O’Rourke et al. 2018). A complete and recorded OBTT thus provides all those involved in the care of a woman in labour with a clinical picture as well as a care plan (Baker et al. 2015). An OBTT thus ensures continuity of care and prevents repetition of nursing care, thus preventing medicolegal hazards (Van Graan, Williams & Koen 2016). The condition of a woman in labour is detailed, and a classification based on the existing and potential problems is carried out, which helps to reduce the waiting time (Marombwa et al. 2019). An increase in waiting time has been associated with a higher rate of negative perinatal outcomes as the clinical conditions are worsened (Alharbi et al. 2018). The ability of a complete OBTT to ensure a timeous and standardised plan of care is executed, and it is associated with an increase in positive pregnancy outcomes (Marombwa et al. 2019).

## Research purpose

This study explored and described midwives’ experiences of the OBTT used during admission of women in labour in the Bojanala district.

## Research design

A qualitative, explorative, descriptive research design was employed to uncover the midwives’ experiences of the OBTT used during admission of women in labour.

### Population and sampling

Midwives were invited to participate in the study from the population of midwives employed in the two selected midwifery care-rendering facilities in the Bojanala district. Purposive sampling methods using an inclusion criteria were used to select the prospective participants. In order to meet the inclusion criteria, the midwives were to be full-time employees with at least 5 years of clinical midwifery experience in the Bojanala district health facilities.

### Sampling process

Twelve prospective participants who met the inclusion criteria were invited to participate in the study through their operational managers identified as gatekeepers. Nine midwives (Table 1) showed interest in the study and made telephonic contact with the researcher to set an appointment convenient for them in terms of date, time and venue. The calculated response rate was 75%. The researcher made the participants’ contact numbers inaccessible to others, guided by the *Protection of Personal Information Act* (POPIA). The participants opted to conveniently be interviewed in their respective homes and stated that it is impossible for them to take time off their midwifery responsibilities. The researcher honoured the appointments and drove to each participant’s home.

**TABLE 1:** Summary of description of the participants.

Code	Age	Gender	Ethnicity	Qualifications	Experience
P1	27	Female	Black	Bachelor of Nursing & Midwifery	5 years
P2	40	Female	Black	Diploma in Nursing & Midwifery	16 years
P3	42	Female	Black	Diploma in Nursing & Midwifery	8 years
P4	52	Female	Black	Diploma in Nursing & Midwifery Diploma in Advanced Midwifery	18 years
P5	27	Female	Black	Diploma in Nursing & Midwifery	5 years
P6	40	Female	Black	Diploma in Nursing & Midwifery Diploma in Advanced Midwifery	12 years
P7	40	Female	Black	Bachelor of Nursing & Midwifery	16 years
P8	26	Female	Black	Bachelor of Nursing & Midwifery	5 years
P9	36	Female	Black	Diploma in Nursing & Midwifery	7 years

### Data collection

The complete approvals to collect data were received at the end of January 2019. Semistructured interviews were conducted between February 2019 and May 2019 as a mode of data collection. The interviews lasted between 30 min and 55 min. The researcher obtained voluntary consent for the interviews to be audio-recorded. The following open-ended central question was posed to the participants: ‘What is your experience of the obstetric triage tool?’

The researcher probed into the participants’ responses to gain an in-depth understanding of their experiences of the OBTT. The researcher used minimal responses to allow uninterrupted flow of communication from the participants. Because all the interviews were recorded, the researcher ensured that each interview was transcribed verbatim within 24 h. The immediate transcription ensured that the researcher had recall of all the events. The transcribed data and audio notes were kept in a password-encrypted electronic file, accessible only to the researcher. Data collection was carried out to a point of data saturation, confirmed by repetition of information by the seventh interview. Two more interviews were conducted to validate that there was no emergence of new data.

### Data analysis

Colaizzi’s (1978) seven steps of descriptive data analysis was used to discover the experiences of midwives with regard to the OBTT currently in use (Polit & Beck 2019). The researcher listened to audio recordings and read the verbatim transcriptions with the intention to familiarise himself with the collected data. The researcher made note of data deemed relevant to the research question and the study to formulate underlying meanings. The researcher clustered similar themes and made a detailed description of midwives’ experience of the OBTT with all identified themes. An independent coder, who is an expert in qualitative data analysis, analysed all the transcriptions. A meeting was arranged for independent coder and the researcher to discuss the themes, and consensus was reached.

### Trustworthiness

To ensure trustworthiness, the researcher applied Lincoln and Guba’s (1986) principles of credibility, transferability, dependability, confirmability and authenticity (Polit & Beck 2019). As a measure of dependability, the researcher interviewed participants who met the inclusion criteria as per the approved research protocol, up to the point of data saturation. To ensure credibility, the researcher held interviews lasting between 30 min and 55 min with the participants, which allowed for prolonged engagement with the participants. As a measure to ensure confirmability, the researcher kept an audit trail of all documentation regarding the research study, for example, all audiotaped material, written notes as well as the verbatim transcriptions. The record of field notes was used to clarify the midwives’ experiences of the OBTT, and they formed part of the audit trail regarding the study (Polit & Beck 2019). As a measure to ensure the authenticity of the study, the researcher captured in the text the real feelings, moods, experiences and language as expressed by the participants to maintain the real feel of the context of the study. As a measure to ensure transferability and generalisability of findings, the researcher provided a dense description of the participants’ demographic information related to the study. Midwives’ direct quotations were used during the discussion of data analysis to enrich the description of their experiences of the current OBTT and recommendations for an ideal tool. To strengthen the validity of the research findings, an independent coder was employed to analyse data. A consensus meeting about preliminary and final results was held between the researcher and the independent coder. The researcher presented the research findings to all participants, who confirmed that the findings were descriptive of their experiences.

### Ethical considerations

The researcher made it a point to adhere to all the ethical considerations prescribed for health science research. Ethical clearance to conduct this study was obtained from the University of Johannesburg Research Ethics Committee, reference number: REC-01-172-2018. The study was registered and approved by National Health Research Database (NHRD). Approval was also granted by the North West Department of Health for access to participants in the Bojanala district health facilities though their managers, identified as gatekeepers.

In respect of participants’ right to autonomy, detailed information pertaining to the study was provided to gain their voluntary and informed consent to participate in the study. The researcher emphasised that participants were free to withdraw from the study at any given point to eliminate feelings of coercion and encourage free expression. To maintain participants’ privacy, confidentiality and anonymity, the researcher generated codes specifically for data collection, analysis and discussion. Furthermore, the researcher limited discussion of data to self, supervisors and independent coder. Audio recordings, transcriptions and field notes were kept in a password-encrypted file.

## Results

One major theme detailed in (Table 2) emerged as follows: the midwives experienced the current OBTT as deficient in the collection of comprehensive clinical data on admission due to space constraints, compromising the quality of midwifery care, which led to midwives’ recommendations for its revision. Ten subthemes emerged, comprising midwives’ verbalised dissatisfaction with the OBTT and their recommendations for revision of the OBTT to have adequate spaces to capture all the findings (Table 2).

**TABLE 2:** Summary of description of themes and subthemes.

Theme	Subthemes
1. The midwives experienced the current OBTT to be deficient in collection of a comprehensive clinical data on admission due to space constraints, compromising the quality of midwifery care, which led to midwives’ recommendations for its revision	Adequate space for vital signs. Adequate space for recent blood results. Adequate space for high-risk conditions. Adequate space for abdominal examination findings. Adequate space for admission cardiotocograph findings Adequate space for initial per vaginal examination findings. Adequate space for comprehensive physical and psychological examination. Adequate space for admission sonar. Adequate space for comprehensive admission report and care plan. Adequate space for diagnosis and classification.

OBTT, obstetric triage tool.

### Adequate space for vital signs

Midwives mentioned that they need an adequate space to record all of the vital signs they monitor on a woman during OBT for diagnostic purposes. They said:

‘[*W*]hen we triage patient[*s*], we are supposed to do all the vital signs, which means we will have to check the BPs [*blood pressure*], the HGTs [*haemoglucotest*] ….’ (P3, Midwife, 8 years’ experience)

‘But there is no space to document it ….’ (P1, Midwife, 5 years’ experience)

They recommended that the space for vital signs be increased to record all the details of such vital signs:

‘I think they should amend the initial assessment of labour, especially with the vital data … the glucose is not there ….’ (P4, Midwife, 18 years’ experience)

‘They should have the column of vital signs! For pulse, BP, temperature and respiration ….’ (P9, Midwife, 7 years’ experience)

### Adequate space for recent blood results

The midwives recognise the importance of routine and special investigations in directing the plans of care. However, midwives experienced that there are no designated spaces to write all the results of the routine rapid blood results:

‘There are no spaces to write rapid results such as Rh or HB [*haemoglobin*]; they only stated the booking HB.’ (P7, Midwife, 16 years’ experience)

Midwives acknowledge the effects of the physiology of pregnancy on the haemoglobin level of the woman and recommended that there be a space to record haemoglobin tested on admission:

‘When you admit the patient, you must write the current HB ….’ (P7, Midwife, 16 years’ experience)

Furthermore, the midwives recognised the progress in HIV management and noted the fact that the prescribed drugs according to the OBTT are outdated:

‘About the HIV status of the patient! The book has not yet been modified; we are no longer using AZT [*azidothymidine*].’ (P4, Midwife, 18 years’ experience)

Midwives recommended that there be spaces for recording of all the routine investigation findings:

‘But where must we record the HB on admission? We need space for it.’ (P7, Midwife, 16 years’ experience)

### Adequate space for high-risk conditions

Midwives are aware of all the existing risk factors and their possibility of bringing about complications. Midwives complained that there is no designated space to indicate the pre-existing risk factors to alert the receiving midwives of the woman’s condition:

‘You find that the patient is a high-risk patient or is having more than one risk factor, so the space is not enough, so we are limited to write everything.’ (P6, Midwife, 12 years’ experience)

The midwives recommended that there be space made available to write the risk factors to instantly alert the midwives of the patient’s condition:

‘I think this … tool that we are using should be revised and then all the information be included in that tool and give enough space to write everything.’ (P6, Midwife, 12 years’ experience)

### Adequate space for abdominal examination findings

Midwives explained their value for abdominal examination performed during admission and the relevance of findings of such an examination:

‘When the patient comes in, you will want to do the abdominal examination to assess the contractions and the size of the foetus ….’ (P1, Midwife, 5 years’ experience)

Midwives vehemently expressed that it seemed as though the tool does not appreciate the midwives’ skills and knowledge in the diagnosis of true labour:

‘On the initial assessment they have got these contractions: yes, no, unsure … I mean, as a midwife, I cannot be unsure of contractions ….’ (P4, Midwife, 18 years’ experience)

Midwives were unimpressed with the space provided for the recording of abdominal examination findings on OBTT and made recommendations:

‘If they could just provide us with space to write all our abdominal examination findings so that they make sense to another midwife or the doctor.’ (P5, Midwife, 5 years’ experience)

### Adequate space for admission cardiotocograph findings

The midwives recognise the need for an admission CTG necessary to determine the foetal well-being:

‘On admission, the patient should be assessed and do the tracing of the foetal heart rate.’ (P2, Midwife, 16 years’ experience)

The midwives complained that they have limited space to record CTG findings:

‘And it doesn’t show; it just says “the foetal heart rate” … then normal or abnormal.’ (P7, Midwife, 16 years’ experience)

Midwives recommended that there be adequate space for them to write CTG findings and be able to elaborate on them and make interpretations:

‘I think they must put the space where you would say it was 140 to 160 beats per minute because we say foetal abnormalities ….’ (P7, Midwife, 16 years’ experience)

### Adequate space for initial per vaginal examination findings

Midwives regard the initial vaginal examination as the diagnostic tool for the onset of labour and a baseline for progress of labour:

‘The patient comes complaining of “I am in labour” …. The first thing that you will do is – you will be focusing on is PV [*per vaginal*] ….’ (P9, Midwife, 7 years’ experience)

Midwives revealed that they are knowledgeable about the per vaginal examination (PVE) and related findings. They explained that there are other important findings, and the emphasis is not only on the dilatation of the cervix:

‘[*W*]hen the patient comes, it’s like we are only concentrating on what the OB [*obstetric*] tool is asking us to do; it’s like we are not obtaining all much-needed information from the patient.’ (P9, Midwife, 7 years’ experience)

Midwives revealed that the current OBTT makes it difficult for them to record the findings obtained during PVE for a detailed interpretation by others:

‘There is no space; there is a lot write.’ (P7, Midwife, 16 years’ experience)

Midwives suggested that there should be space for detailed PVE made available for them:

‘Let them create space to include most of the things, like caput, moulding, head level, liquor, etc. ….’ (P4, Midwife, 18 years’ experience)

### Adequate space for comprehensive physical and psychological examination

Midwives recognise the need for performing a comprehensive physical examination, which provides them with a clinical picture of the woman on admission:

‘When you admit the patient, you assess the patient, how was the patient when she got in, but there is no space for it … There is a lot to write ….’ (P7, Midwife, 16 years’ experience)

The midwives complained that there is no adequate space for them to write their physical examination findings, which limits their ability to draw the clinical picture:

‘We can’t even record how the patient was on admission.’ (P6, Midwife, 12 years’ experience)

The midwives recommended space for writing clinical examination findings:

‘I wish they could give us space for those kinds of elaborations ….’ (P3, Midwife, 8 years’ experience)

### Adequate space for admission sonar

Midwives expressed that the admission sonar is indicated for high-risk patients because of its ability to provide diagnostic data:

‘There are patients who really needs sonar, for instance, a patient with APH [*antepartum haemorrhage*] or placenta abruption ….’ (P5, Midwife, 5 years’ experience)

Midwives expressed that the obstetric sonar requires a specialised skill from advanced midwives and doctors. However, midwives expressed the value of obstetric sonar findings in planning their management:

‘Sonar is being done by the doctor and the advanced midwives sometimes ….’ (P7, Midwife, 16 years’ experience)

Midwives noted with concern that there is no designated space for advanced midwives and doctors to detail the obstetric sonar findings for their convenient access:

‘They must write the assessment of the sonar after they had assessed the woman …. but there is just no space for it ….’ (P7, Midwife, 16 years’ experience)

The midwives recommended that there should be space for detailing admission obstetric sonar findings:

‘I wish there was space for sonar as well, especially if the patient is a high risk; we need to see all those findings written down ….’ (P8, Midwife, 5 years’ experience)

### Adequate space for comprehensive admission report and care plan

Midwives seemed to be familiar with the nursing process approach and the need for standardised midwifery care prescribed during OBT:

‘I don’t know how we can manage the patient without planning for the patient …. I must plan for the patient ….’ (P7, Midwife, 16 years’ experience)

Midwives understand that the clinical notes and care plans need to be detailed and sequential in nature:

‘Even when you go to management, there is a lot to write about; most of the things are not included there ….’ (P7, Midwife, 16 years’ experience)

Midwives revealed that the lack of space poses a risk of causing the fragmentation of clinical information and possible loss of such valuable information:

‘In the summary side, it is – there is no space because there is a lot write. Which causes us to go to another page, like when you … most of the things are not included there ….’ (P7, Midwife, 16 years’ experience)

The midwives were adamant that the loss of such information interferes with the continuity of care and it is detrimental to the quality of care to be received by the woman:

‘So now it will be missing other important information; other sisters will not know what you did to the woman ….’ (P5, Midwife, 5 years’ experience)

Midwives described what they believed will serve as an adequate space for writing a comprehensive admission report and nursing care plans:

‘I would like them to give us more space to write the management as it is important as we are transferring the patient to another higher-level hospital … they will see what you did when they take over ….’ (P6, Midwife, 12 years’ experience)

### Adequate space for diagnosis and classification

Obstetric triage findings lead to diagnosis and classification of a woman, and the midwives regard this as important. The midwives affirm that the diagnosis and classification of a woman during OBT is important in alerting them of existing and potential obstetric problems. However, midwives reported that the current OBTT does not have a designated space to detail the classification and diagnosis. They said:

‘There is no, like, a tick box or something that will be convenient just to make you alert that this person is having a medical condition.’ (P2, Midwife, 16 years’ experience)

The midwives then made a recommendation for the incorporation of the designated spaces for the diagnosis and the classification of the woman:

‘They could just say medical condition … tick yes, and then specify, which might be easier because they can’t list all the medical conditions that the patient might have.’ (P1, Midwife, 5 years’ experience)

## Discussion

The study findings highlighted the midwives’ concerns about the current OBTT. The said concerns were followed by the descriptions of what midwives believe to be an ideal OBTT. From the analysed data, one central theme and 10 subthemes emerged detailing the midwives’ dissatisfaction with the OBTT and their recommendation to amend the existing tool. A noteworthy finding was a realisation of midwives’ great value for correct and comprehensive record-keeping of obstetric-related clinical information, which substantiates O’Rourke et al.’s (2018) claims that midwives prefer verbal reports to support the written clinical reports. The midwives’ demonstrated interest in record-keeping proved that they carry out their profession as stipulated in the scope of practice (R2488) and maternity care guidelines (DOH 2016).

**FIGURE 1:**
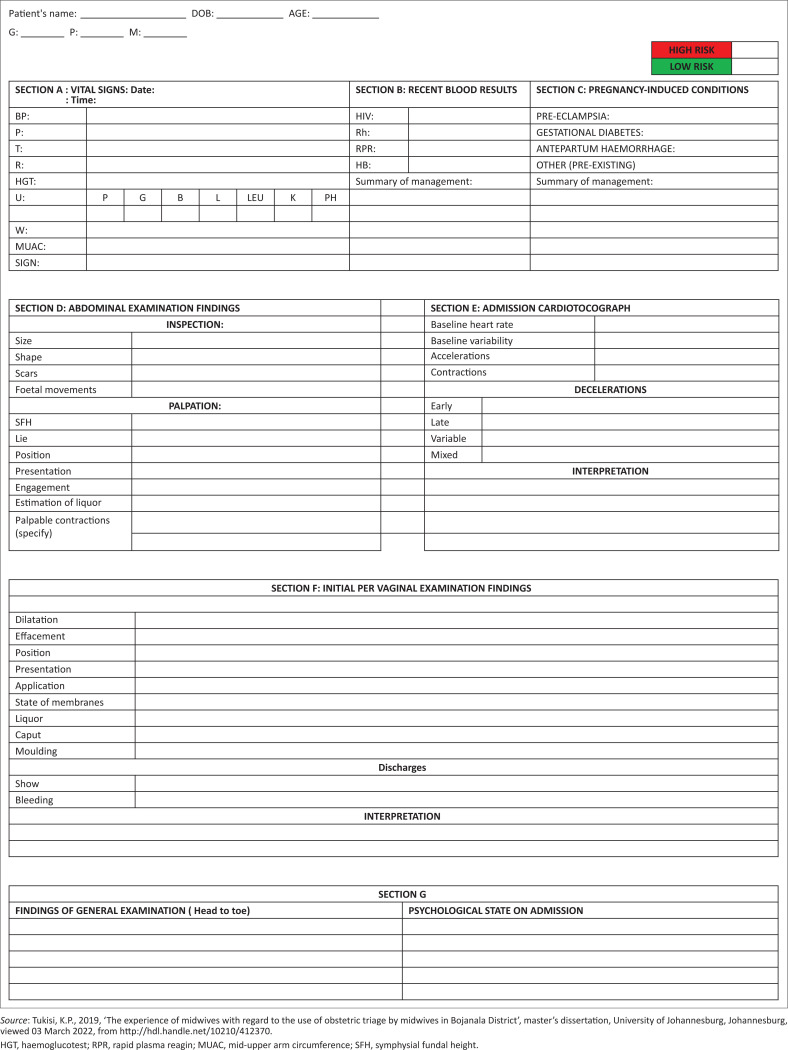

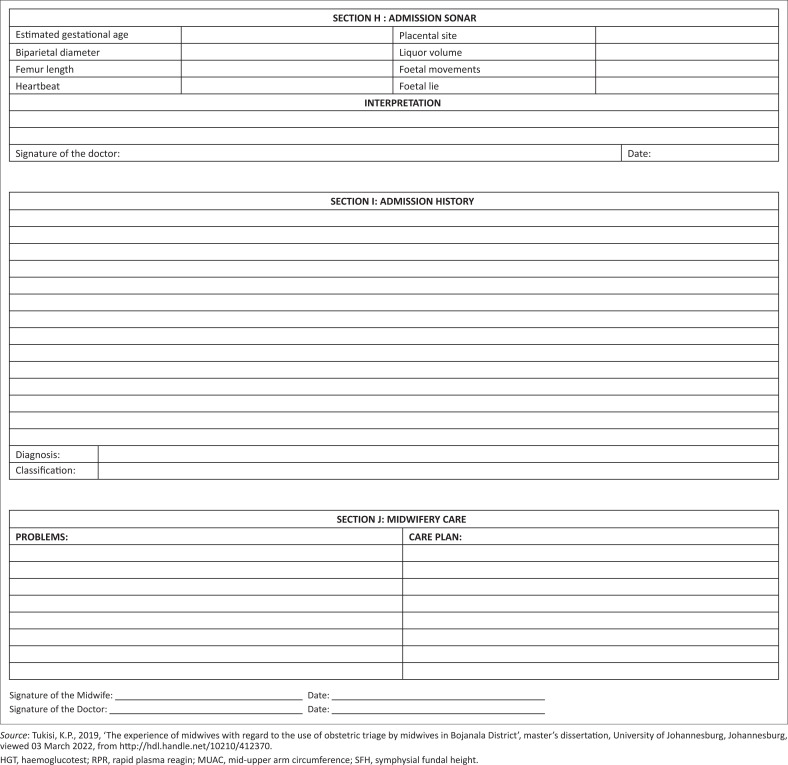
An ‘ideal’ obstetric triage tool.

The midwives’ concern for inadequate space for recording all the admission vital signs in the OBTT is valid as per OBT protocols (Angelini & Lafonte 2017). Admission vital signs form a major part of OBT for diagnostic purposes. It is therefore empirical for the vital data to be recorded to serve as a baseline (DOH 2016). The midwives recommended that there be spaces within the OBTT for recording all vital signs detailed in Figure 1: Section A. The midwives argued that vital signs are diagnostic, and they intend to diagnose medical conditions immediately on admission. This finding concurs with the findings from Nathan et al. (2018) that life-threatening conditions such as pre-eclampsia are detectable throughout pregnancy, even during labour, by monitoring blood pressure and performing urinalysis. The midwives insisted that pulse and temperature should be recorded on admission; this is significant as it indicates midwives’ intention to exclude and treat chorioamnionitis, which could lead to foetal distress (Charpentier et al. 2022). The midwives proposed that space be provided to record the haemoglucotest (HGT), weight and mid-upper arm circumference performed during admission; this is crucial as it indicates that the midwives critique the prescribed guidelines on maternity care which limits these assessments to basic antenatal care (ANC) (DOH 2016). This finding proved the midwives’ knowledge of the ongoing physiology of pregnancy and possible onset of gestational diabetes and maternal weight gain that could lead to foetal macrosomia, thus influencing the choice of delivery methods (Nguyen & Ouzounian 2021). This finding demonstrated midwives’ application of critical thinking and clinical judgement to address clinical situations, as it is a requirement for nursing and midwifery practice (Jessee 2021).

Inadequate space for documentation of recently collected pregnancy routine blood tests was among the midwives’ concerns. The midwives recommended that there be spaces to record rapid and recent routine blood investigation detailed in Figure 1: Section B. Midwives insisted that it is imperative for these blood tests to be collected from all pregnant women in labour and recorded in the OBTT. The midwives based their argument on their knowledge of physiological changes taking place during pregnancy. Midwives revealed that the haemoglobin reading is altered throughout pregnancy by the increase in the plasma levels by almost 40%, causing haemodilution, according to Sellers, Dippennaar and Da Serra (2018); therefore, it is not clinically sound to rely on a haemoglobin reading recorded during ANC (Morrison et al. 2021). The midwives seemed aware of the possibility of foetal distress and postpartum haemorrhage secondary to the presence of anaemia, as indicated by Sellers et al. (2018), and insisted that a record of an admission haemoglobin should be made on the OBTT. Midwives were adamant that there should be a designated space on the OBTT to record rapid HIV and syphilis tests on the woman on admission during labour. This particular finding concurs with Ainslie and Therivel’s (2021) claims that HIV and syphilis are rife among pregnant women, and this is because the state of pregnancy eliminates the fear of unprotected sex, thus increasing chances of cross-infection.

Midwives pointed out their concern for insufficient space to record existing high-risk conditions on the OBTT. They alluded that the OBTT, as it is, fails to alert a receiving midwife of the seriousness of the condition of the woman in labour. The midwives recommended spaces within the OBTT to record existing conditions and high-risk factors, detailed in Figure 1: Section C. The midwives elaborated that the existing risk factors will require specific attention and will therefore need to be detailed in the OBTT. This is significant as the midwives are cognisant of the need to tailor the plan of care based on the problem list of the woman in labour. In cases such as the woman suffering from antepartum haemorrhage, the woman will need to be prioritised in order to maximise the chances of survival of both the mother and the foetus (Long et al. 2021).

Another finding that revealed midwives’ dissatisfaction with the OBTT is the standard abdominal examination. Midwives described that they experienced limitations in documenting abdominal examination findings. The midwives recommended spaces to record the abdominal examination findings detailed in Figure 1: Section D. Midwives revealed that they needed to record the progress of pregnancy and insisted that there be space for capturing both abdominal inspection and palpation findings. This concurs with Sellers et al. (2018) that in order to correctly diagnose the foetal position, lie inside the uterus and possibility of engagement inside the uterus, the examiner need to correlate findings from both abdominal inspection and palpation. This finding stresses the midwives’ value of the abdominal examination to determine foetal size and position and its ability to aid them in making choices for the mode of delivery (Rawal, Katuwal & Shrestha 2020).

Another important finding that forms part of the abdominal examination findings is the monitoring of the foetal heart rate (Sellers et al. 2018). The midwives complained limited space to record the detailed report on foetal well-being, importantly the admission CTG findings. The midwives recommended spaces to record and interpret the admission CTG findings, detailed in Figure 1: Section E. This finding lends support to the existing literature stating that an admission CTG can reveal ongoing foetal distress and the early warning signs of anticipated foetal distress (Kale 2022). The findings and diagnosis revealed on the CTG trace can aid with the diagnosis of foetal heart rare abnormalities and necessitate that the foetus be vigilantly monitored (Kale 2022). Such recorded findings can be used to alert the midwives who will be receiving the woman after OBT, which will ensure continuity of planned care. The midwives’ recommendation for recording of CTG findings from all pregnant women being admitted for labour is in contrast with the earlier findings from the Devane et al. (2017), which found that CTG tracing increases caesarean section by almost 20% and recommended the use of CTG tracing only on high-risk women in labour.

Per vaginal examination is significant in midwifery practice (DOH 2016). Midwives complained of limited spaces for them to elaborate on the findings of this assessment when they record on the OBTT. The midwives recommended spaces to record PVE findings detailed in Figure 1: Section F. Midwives emphasised that their recommendation is based on their knowledge of necessary findings a midwife need to be on the lookout for in order to diagnose true labour and monitor its progress. This finding is particularly important as it demonstrated midwives’ value for safer normal vaginal delivery, which could reduce a caesarean section rate as supported by (Jiang et al. 2018).

The finding from this study suggesting that midwives are concerned about the space for recording the psychological status of women in labour is noteworthy. The midwives pointed out that the focus is on the physical examination findings while the psychological status is not addressed. The midwives recommended spaces to record both physical and psychological status assessment findings as detailed in Figure 1: Section G. This finding is a demonstrated willingness on the midwives’ part to nurse the woman in labour holistically, as per the suggestion by Mathibe-Neke and Mondell (2017) that midwifery and nursing care should address the four dimensions, namely the physical, psychological, emotional and spiritual being. Midwives demonstrated an understanding of the need to establish the woman’s psychological status to exclude existing mental health issues, which could be warning signs for possible postpartum blues and depression. A record of psychological problems could alert the midwives to draw up a tailor-made care plan to support the woman during labour (Mathibe-Neke & Mondell 2017). This finding highlights a breakthrough in the midwifery field, where perinatal mental health has been a neglected topic, and provides a positive feedback on the work done by Lewis et al. (2021) to educate midwives on the management of perinatal mental health issues.

Midwives expressed that they are also carrying out OBT of high-risk women, who at times require an ultrasound to confirm the diagnosis. However, midwives expressed that there is no space for recording ultrasound findings. Midwives recommended space within the OBTT, as detailed in Figure 1: Section H. Midwives appreciate that doctors and experienced midwives perform the sonar; this is progressive as it highlights the midwives’ value of obstetric sonar for diagnostic purposes. This may herald a need for revisiting midwifery practice regulations to provide in-service trainings on obstetric sonography to equip midwives with this diagnostic skill (Bwanga, Mwase & Kaunda 2021).

History-taking during OBT plays a pivotal role in the identification of problems, diagnosis and directing the formulation of a midwifery care plan for the woman in labour. The midwives expressed that they lack space in the current OBTT to document the admission history and midwifery care plan during OBT. The midwives recommended spaces to record the admission history and a detailed midwifery care plan, as shown in Figure 1: Sections I and J. Midwives based this recommendation on an argument that upon admission, very little is known about the woman in labour. An OBT history-taking allows midwives to familiarise themselves with the clinical information of the woman in their care; the midwives were adamant that such information needs to be documented on the tool (Kaur et al. 2021). Midwives also revealed that once all the information has been collected and the examination is concluded, a detailed midwifery care plan will need to be formulated and documented, as it will be followed by all those who will care for the woman. This finding is encouraging as it sheds light into midwives’ application of the nursing process approach by following the steps of assessment, diagnosis, planning, implementation and evaluation (Veit-Rubin et al. 2017). The midwives insist on the space for recording a midwifery care plan which highlights the midwives’ adherence to standardised and continuous midwifery care. The midwives’ intent to document the subjective and objective history as well as care plans is intriguing, as it demonstrates the midwives’ awareness of the fact that they are not nursing women in isolation; there is a need for handover of the woman from the OBT room to the progress of labour and delivery rooms (O’Rourke et al. 2018). This handover will require both written and verbal reports, and the midwives’ eagerness to record is encouraging, corroborating the findings of a study by O’Rourke et al. (2018) that documentation limits the room for miscommunication of clinical information, which can negatively influence standardised care.

## Conclusion

This study has summed up the midwives’ description of an ideal OBTT. The findings indicated the midwives’ eagerness to improve the current OBTT in use based on their recommendations. A qualitative and descriptive research design provided a full description of an ‘ideal OBTT’ which could be of diagnostic value in OBT settings, as found in this study.

The findings suggested that there is a need to alter the existing OBTT to make it more comprehensive for the recording of all clinical data gathered during OBT. Midwives were in the forefront to make recommendations, and their recommendations are clinically sound and well substantiated by the existing literature on OBT. The researcher is also of the opinion that an improved OBTT as per midwives’ recommendations could be of aid in the early detection and diagnosis of obstetric-related problems because of the availability of comprehensive clinical data. The researcher also believes that the tool could be useful in increasing positive pregnancy and labour outcomes.

The findings led the researcher to devise an ‘ideal OBTT’ which could add value in clinical practice based on the midwives’ recommendations and the existing literature (see Figure 1). The designed tool could provide a framework of reference to improve the current OBTT in use.

The following limitations were identified. The study focused on midwives’ experiences and excluded the doctors working very closely with midwives in the OBT setting, and they could have provided insightful recommendations for the OBTT. The described tool has not yet received evaluation by the midwifery experts for relevance to nursing education, research and practice. On a positive note, the ‘ideal OBTT’ is under study for revision and evaluation by a team of experts.
